# Comparison of radiological features of high tibial osteotomy and tibial condylar valgus osteotomy

**DOI:** 10.1186/s12891-019-2764-0

**Published:** 2019-09-04

**Authors:** Takashi Higuchi, Hironobu Koseki, Akihiko Yonekura, Ko Chiba, Yusuke Nakazoe, Shinya Sunagawa, Chieko Noguchi, Makoto Osaki

**Affiliations:** 1 Department of Health Sciences, Nagasaki University Graduate School of Biomedical Sciences, 1-7-1 Sakamoto, Nagasaki, 852-8520 Japan; 2 Institute of Biomedical Sciences, Nagasaki University, Nagasaki, Japan; 3 Department of Orthopedic Surgery, Nagasaki University Graduate School of Biomedical Sciences, Nagasaki, Japan; 4 Department of Orthopedic Surgery, Wajinkai Hospital, Nagasaki, Japan

**Keywords:** Knee osteoarthritis, High tibial osteotomy, Tibial condylar valgus osteotomy

## Abstract

**Background:**

The purpose of this study was to compare radiological features between high tibial osteotomy (HTO) and tibial condylar valgus osteotomy (TCVO), in order to define the radiological indication criteria for TCVO.

**Methods:**

Thirty-two cases involving 35 knees that had undergone HTO and the same number that had undergone TCVO for knee osteoarthritis were retrospectively evaluated. Characteristics of both groups did not differ significantly. Lower limb alignment, bone morphology, joint congruity, and joint instability were measured in standing full-length leg and knee radiographs obtained before and after surgery.

**Results:**

Radiological features in the TCVO group included greater frequencies of advanced knee OA grade, varus lower limb malalignment, depression of the medial tibial plateau, and varus-valgus joint instability compared to the HTO group before surgery. However, tibial morphology, alignment of the lower limb, and joint instability improved to comparable levels after surgery in both groups.

**Conclusions:**

TCVO appears preferable in cases with advanced knee OA, destroyed or inclined medial tibial plateau, widened and subluxated lateral joint, and high varus-valgus joint instability.

## Background

Knee osteoarthritis (OA) is one of the most common musculoskeletal disorders, especially among the elderly [1–3]. About 8 million and 25 million individuals are affected by symptomatic and asymptomatic knee OA, respectively, in Japan [4]. Surgical approaches to the treatment of advanced medial unicompartmental knee OA have received considerable attention, and recent studies have highlighted the efficacy of osteotomy and prosthetic arthroplasty [5–7]. Due to advances in both materials and designs, the longevity of total knee arthroplasty (TKA) has increased, and patients from a diverse age range are now undergoing this procedure [6, 7]. However, TKA has some problems with material durability, the risk of metal allergies and patient dissatisfaction with joint range of motion (ROM), especially in young, physically active patients [8–10]. Moreover, concerns have been raised regarding complications such as deep or superficial implant-associated infections, wear of the prosthesis, and vein thromboembolism [11–13]. Therefore, osteotomy procedures have been recommended for young and physically active patients wanting to maintain wide ROM, or for individuals who participate in high-demand activities and want to avoid prosthetic arthroplasty [14, 15]. Open-wedge high tibial osteotomy (HTO), the most common osteotomy procedure for treating knee OA [15, 16], is based on the concept of realignment to redistribute weight-bearing and mechanical stress laterally to areas with less destruction, thus relieving pain and improving function [16]. As tibiofibular joint disruption and peroneal nerve injury are potential complications associated with lateral closed-wedge HTO, the medial-approach open-wedge HTO, which avoids such complications, has gained popularity [17–19]. Recent developments in internal fixator devices, surgical techniques, and artificial bone graft have enabled early bone union and gap filling, contributing to better clinical outcomes [20]. Even in open-wedge HTO, however, risks include lateral hinge fracture, damage of neurovascular tissue by long proximal screws, loss of correction, and overcorrection due to implant loosening and nonunion [5, 19, 21]. Furthermore, negative effects on the patellofemoral (PF) joint, limited knee extension, and disease progression due to ligamentous joint laxity remain a concern [22–24]. Knee OA with a Kellgren-Lawrence (K/L) grade [25] ≥ 2 or laxity of the knee joint represent risk factors for declining clinical outcomes after HTO [24, 26]. Hence, in terms of indications, HTO is restricted to patients with mild to moderate medial knee OA in which high joint stability is maintained [5, 15].

Tibial condylar valgus osteotomy (TCVO), a novel L-shaped osteotomy developed in the 1990s in Japan, also corrects lower extremity alignment from varus to valgus and shifts the weight-bearing (mechanical) axis laterally [27]. TCVO together with remodeling of the shape of the tibial plateau can improve femorotibial joint congruity and stability. The combined features of osteotomy and arthroplasty are thus promising for effective treatment of severe knee OA [28]. Due to improvements in implants in recent years, TCVO is now making use of locking plates, resulting in shorter postoperative rehabilitation. In our institute, HTO and TCVO are selected individually on a case-by-case basis for medial knee OA and have yielded almost all successful results [27]. However, TCVO is not widespread because of the technical difficulties and uncertain universal radiological indications. To date, no studies have investigated radiological features of TCVO compared to HTO, and radiological indication criteria for TCVO have not been identified.

The purpose of this study was to evaluate differences in radiological features between HTO and TCVO in detail, and to clarify the radiological indications for TCVO, to facilitate decision-making when choosing between the two surgical techniques.

## Methods

### Subjects

A total of 64 cases involving 70 knees that had undergone osteotomy in our institute from May 2008 to January 2016 were retrospectively evaluated and included in our study. The indication for osteotomy was medial unicompartmental knee OA in relatively young patients (< 65 years of age) and physically active high-demand individuals with near-normal lateral femorotibial compartment, ROM > 90° and flexion contracture < 10°. Patients with lateral OA, advanced patellofemoral arthritis, lateral bowing of the femur, inflammatory arthritis (such as rheumatoid arthritis), or current smoking status were excluded from osteotomy surgery. In particular, OA knees with high varus-valgus joint instability, depression or inclination of the medial tibial plateau (Pagoda deformity [29]), lateral joint dilation, and lateral tibial thrust > 1 cm were included for TCVO, whereas other cases with high joint stability and without depression of the medial tibial plateau were included for HTO, in accordance with the criteria of the International Society of Arthroscopy, Knee Surgery and Orthopedic Sports Medicine (ISAKOS) [15]. The HTO group comprised 32 cases (35 knees) that had undergone HTO. The TCVO group comprised 32 cases (35 knees) that had undergone TCVO. No significant differences in background characteristics were apparent between the two groups (Table 1). The present study was approved by the research ethics committee at Nagasaki University Graduate School of Biomedical Sciences (approval number 2015–15082031), and all patients provided their written informed consent to participate and approved the publication of their data.

**Table 1:** Characteristics of subjects

	HTO group	TCVO group
Age (years)	58.3 ± 8.4	58.4 ± 8.1
Sex (cases/knees)		
Men	17 / 19	16 / 17
Women	15 / 16	16 / 18
Side (knees)		
Right	18	15
Left	17	20
Hight (cm)	161.3 ± 9.4	159.1 ± 8.5
Weight (kg)	72.8 ± 15.8	70.8 ± 13.8
BMI (kg/m^2^)	27.9 ± 5.3	27.8 ± 4.2

### Surgical procedure

The correction angle was estimated by preoperative planning using anteroposterior long-leg weight-bearing radiographs and finally determined by the alignment rod connecting the hip center to the ankle center intraoperatively, aiming to achieve around 62% of the weight-bearing line percentage in both osteotomy methods [30, 31]. The patient was placed in the supine position on a radiolucent operating table and a tourniquet was applied. Initial arthroscopy was performed to document medial-compartment arthritis and to assess the status of the lateral and patellofemoral compartments and menisci.

#### HTO

Biplanar open wedge osteotomy was performed as described by Staubli et al. [30]. A skin incision was made at the proximal tibia through the pes anserinus. Proximal to the pes anserinus, the medial collateral ligament (MCL) was dissected off the posteromedial cortex of the tibia and a blunt Hohmann retractor was inserted to protect the neurovascular structures. Two guide wires were inserted at a point 3.5–4 cm below the medial joint line and passed obliquely 1 cm below the lateral articular margin of the tibia towards the tip of the fibular head. The first osteotomy was performed distal to the guide wires to the upper position of the proximal tibiofibular joint. The osteotomy was incomplete, leaving 10 mm of lateral cortex intact, referred to as the bone bridge, to serve as a hinge point during opening of the osteotomy. The second frontal osteotomy plane started in the anterior one-third of the proximal tibia at an angle of 100° to the first osteotomy plane. An osteotomy was gradually opened until the desired, preoperatively determined alignment had been reached. After the planned gap was obtained, the osteotomized gap was filled with two triangular wedged blocks of bone substitute comprising hydroxyapatite with beta-tricalcium phosphate (β-TCP) featuring 60% porosity (Osferion®; Olympus TerumoBiomaterials Corp., Tokyo, Japan). A TomoFix™ plate (DePuy Synthes, West Chester, PA) was placed on the anteromedial aspect of the tibia and a locking screw was inserted. The proximal screws need to be placed deep enough to reach the lateral part of the tibia to support the load.

#### TCVO

**Fig. 1:**
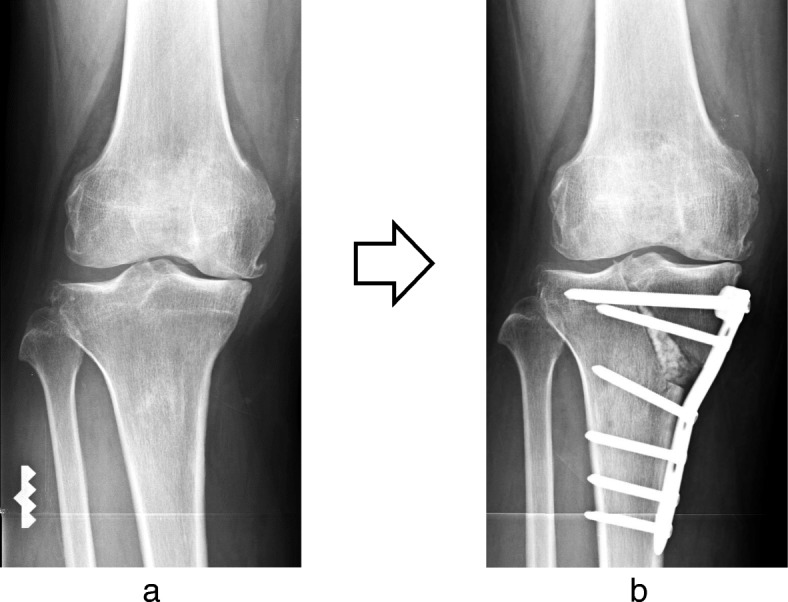
Anteroposterior radiographs of full-length legs in a standing position (**a**) before and (**b**) after TCVO. The L-shaped osteotomy is opened and fixed with TomoFix™ plate. The opened gap space was filled with granular β-TCP

The pes anserinus and superficial layer of the MCL were dissected subperiosteally through a curved skin incision placed distomedially from the medial aspect of the tibial tuberosity. The L-shaped osteotomy was implemented at the medial tibial tuberosity as the apex and extended towards the lateral intercondylar eminence vertically and proximal medial tibia horizontally. Mild valgus force was applied to the leg, and completion of the osteotomy was confirmed on intraoperative radiographic imaging. A Kirschner wire was inserted and stoppers were attached to both ends to prevent separation of the tibial plateau. The osteotomy was opened with gradual valgus force until the desired, preoperatively determined alignment had been achieved. After the correction, a TomoFix™ plate was affixed to the anteromedial aspect of the tibia using locking screws. Granular β-TCP was used to fill the opened gap space (Fig. 1).

### Radiological evaluations

Pre- and postoperative standardized anteroposterior radiographs of full-length legs in a standing position were taken with the feet in a neutral position. Radiographs of the knee joint and manual varus-valgus stress radiographs were also obtained and used for the following measurements.

**Fig. 2:**
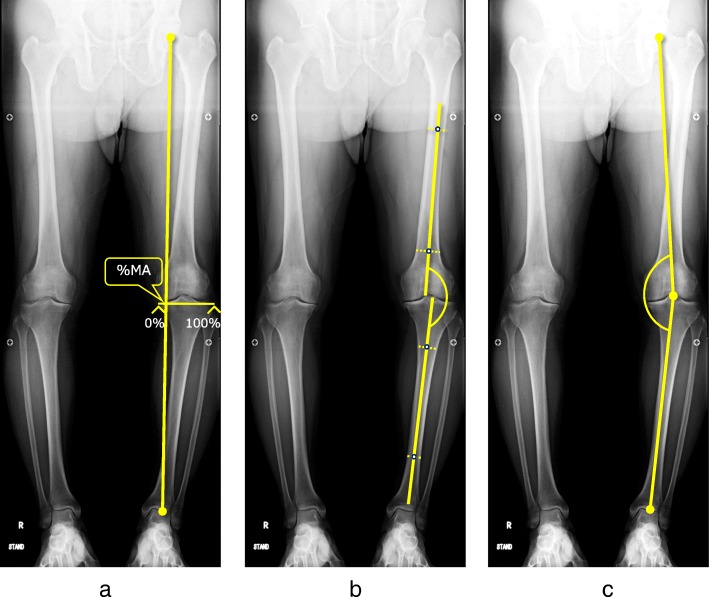
**a**: Percentage of mechanical axis (%MA), **b**: femorotibial angle (FTA), and **c**: hip-knee-ankle angle (HKA angle) were measured to evaluate leg alignment

**Fig. 3:**
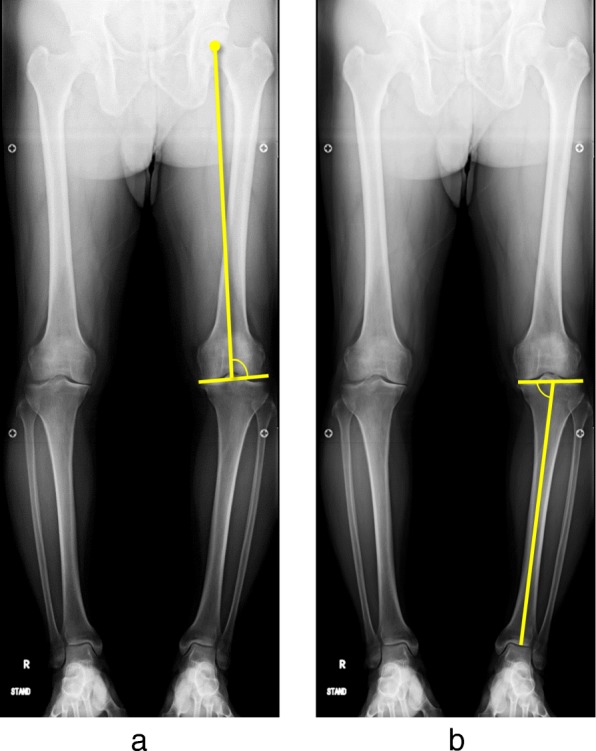
**a**: Mechanical lateral distal femoral angle (mLDFA) and **b**: medial proximal tibial angle (MPTA) were measured to evaluate the morphologies of the distal femur and proximal tibia

**Fig. 4:**
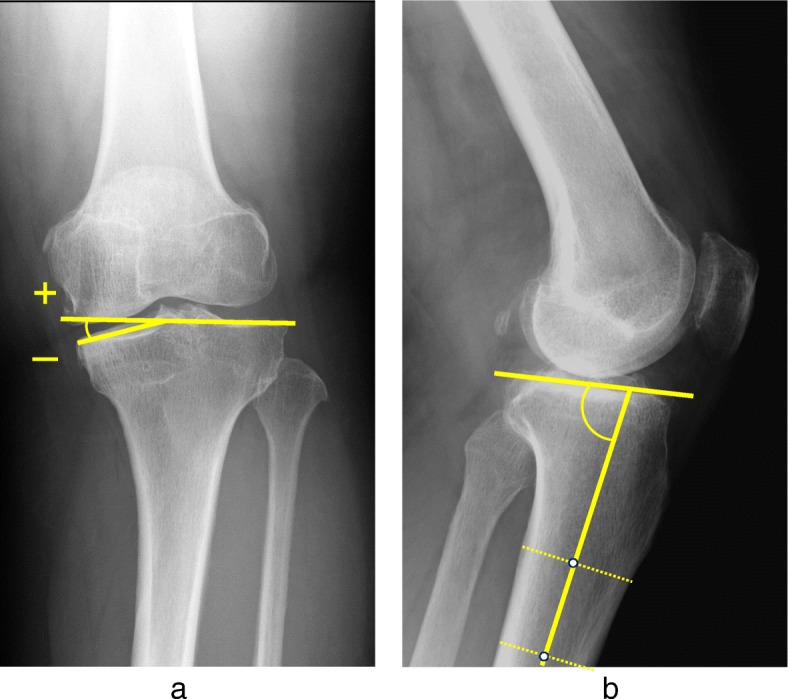
**a**: Medial tibial plateau depression (MTPD) and **b**: Posterior proximal tibial angle (PPTA) were measured to evaluate the morphology of the tibial plateau

**Fig. 5:**
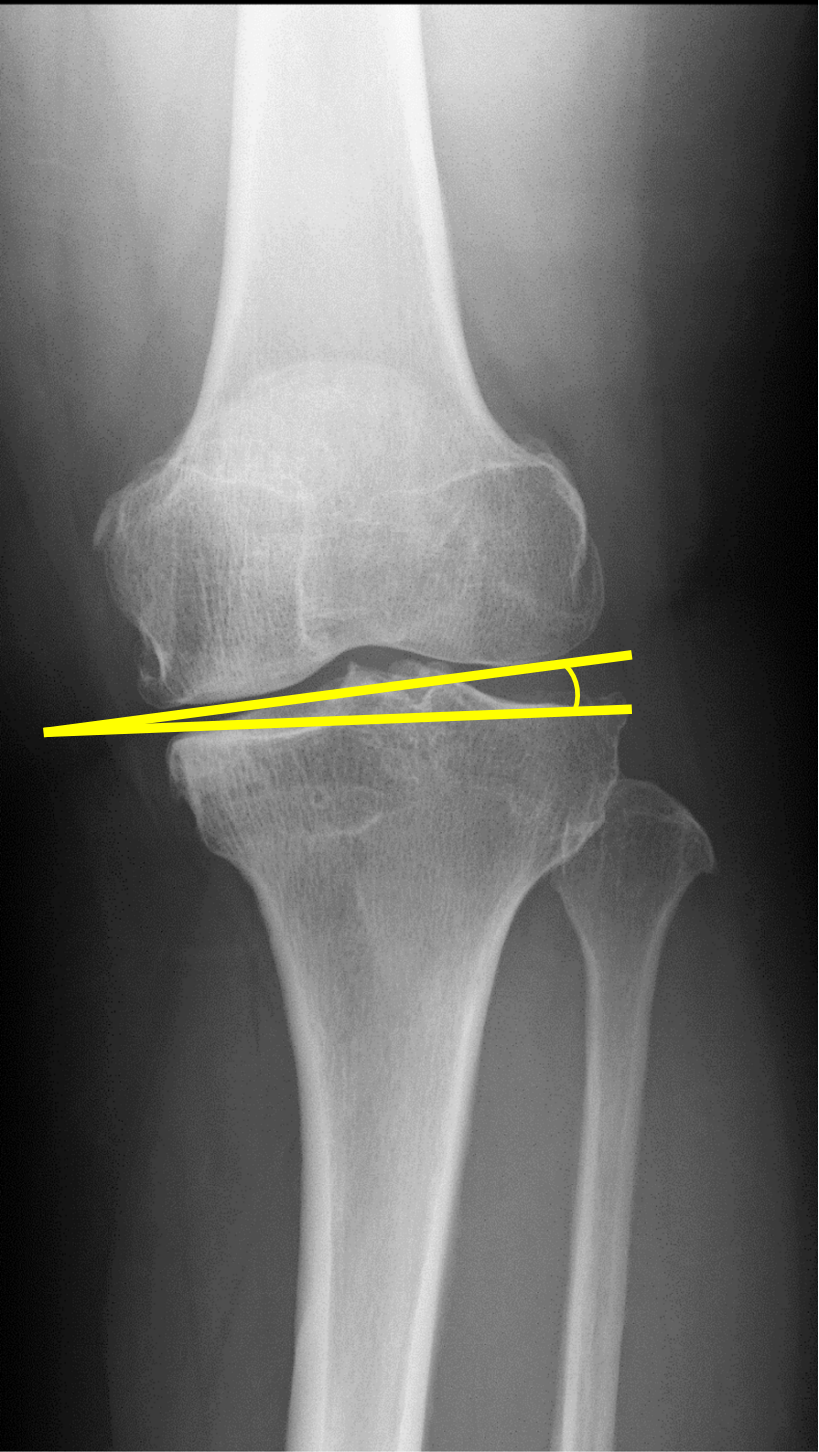
Joint line convergence angle (JLCA) was measured to evaluate knee joint congruity

**Fig. 6:**
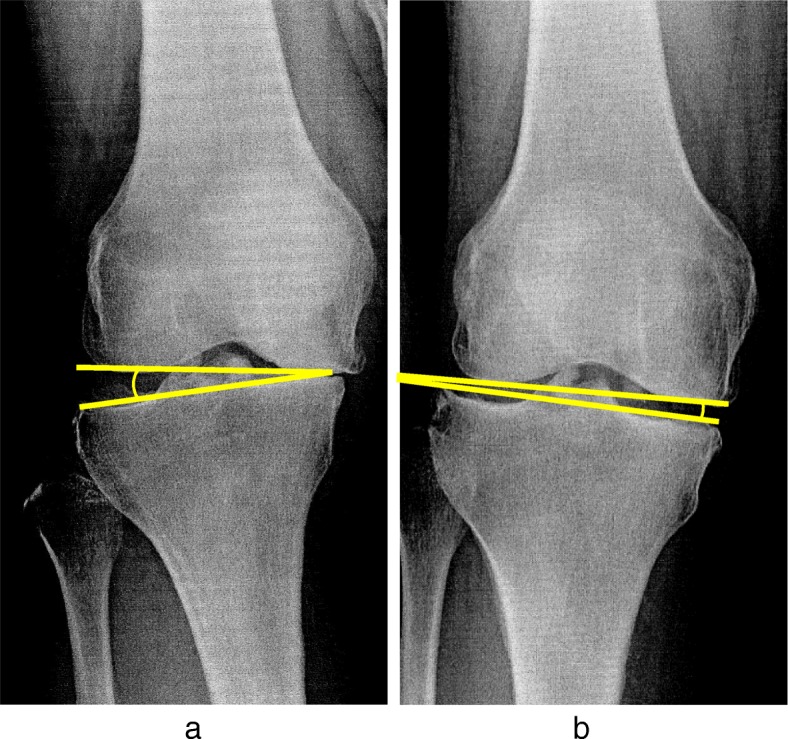
Varus and valgus stress angle. **a**: Varus and **b**: valgus stress were applied and the total amplitude of varus- and valgus-stress angle was identified as the laxity angle

K/L grade was used for classifying the severity of knee OA. The mechanical axis (percentage of mechanical axis: %MA), femorotibial angle (FTA), and hip-knee-ankle angle (HKA angle) were measured to evaluate lower limb alignment (Fig. 2a-c). The %MA indicates the point of intersection between the mechanical axis (a line drawn from the center of the femoral head to the center of the ankle) and the tibial plateau, converted to a percentage from medial edge (0%) to lateral edge (100%) [27, 32]. The mechanical lateral distal femoral angle (mLDFA) and the medial proximal tibial angle (MPTA) were measured to evaluate the morphology of the distal femur and proximal tibia (Fig. 3a, b). Medial tibial plateau depression (MTPD) [33] and posterior proximal tibial angle (PPTA) were also measured to evaluate the morphology of the tibia plateau (Fig. 4a, b). MTPD represents the angle between a line tangential to the lateral and medial plateau. Joint line convergence angle (JLCA) was measured to evaluate knee joint congruity, as the angle formed between a line tangential to the distal femoral condyle and the tibial plateau (Fig. 5). The JLCA in varus- and valgus-stress radiographs was defined as the varus and valgus stress angle, respectively. Total amplitude of varus- and valgus-stress angle was identified as the laxity angle (Fig. 6). Postoperative knee radiographs were taken immediately and 1 year after surgery, whereas standing full-length leg X-rays could not be taken immediately after surgery. Three observers evaluated radiographs from each patient twice, at a minimum interval of 2 weeks. Intra-observer reliability was assessed based on evaluations by the first author, and inter-observer reliability was assessed based on evaluations between the first and second authors. Readers were blinded to the initial measurements, and mean values were taken as the measured values.

### Statistical analysis

Statistical analysis was performed using SPSS Statistics version 22 (IBM, Armonk, NY). Reproducibility and intra-observer reliability of the measurements were assessed using kappa statistics. The unpaired *t*-test or Mann-Whitney *U*-test was used for comparisons between groups. Paired *t*-tests or one-way analysis of variance (ANOVA) and Bonferroni/Dunn post hoc multiple comparison tests were used for comparisons before and after surgery. Values of *P* < 0.05 were considered significant.

## Results

Test-retest reproducibility and intra-observer reliability did not differ significantly (kappa values, 0.76 and 0.86, respectively). No significant differences were identified among the three examiners (*P* > 0.05). Results of each radiological measurement are shown in Table 2. More advanced knee OA was more frequent in the TCVO group (grade 2, 4 knees; grade 3, 21 knees; grade 4, 10 knees) than in the HTO group (grade 2, 19 knees; grade 3, 15 knees; grade 4, 1 knee) (*P* < 0.01). Pre-operative %MA was significantly lower in the TCVO group (8.7 ± 13.3%) than in the HTO group (20.0 ± 11.2%; *P* < 0.01). In terms of lower limb alignment before surgery, FTA was significantly higher in the TCVO group (183.4 ± 3.9°) than in the HTO group (180.9 ± 3.7°; *P* < 0.01) and HKA angle was significantly lower in the TCVO group (170.2 ± 3.2°) than in the HTO group (172.9 ± 2.9°; *P* < 0.01). No significant differences in mLDFA or pre- or postoperative MPTA were seen between groups, but pre-operative MTPD and PPTA were significantly lower in the TCVO group than in the HTO group. Pre-operative JLCA was higher in the TCVO group (5.1 ± 1.5°) than in the HTO group (1.4 ± 1.5°; *P* < 0.01). Pre-operative varus stress angle and laxity angle were significantly higher in the TCVO group than in the HTO group, but no significant difference in valgus stress angle was identified. Conversely, postoperative MTPD was high, and varus stress angle and laxity angle were low in the TCVO group compared to the HTO group (*P* < 0.05), representing inverted situations from preoperatively.

**Table 2:** Radiological parameters

	HTO group			TCVO group		
	Pre-op	Immediate	1-year post-op	Pre-op	Immediate	1-year post-op
K/L grade (II/III/IV)	19/15/1			4/21/10 ^b^		
%MA	20.0 ± 11.2		65.3 ± 8.6 ^a^	8.7 ± 13.3 ^b^		62.1 ± 7.9 ^a^
FTA	180.9 ± 3.7		169.2 ± 2.8 ^a^	183.4 ± 3.9 ^b^		170.5 ± 3.4 ^a^
HKA	172.9 ± 2.9		184.3 ± 2.2 ^a^	170.2 ± 3.2 ^b^		184.3 ± 3.1 ^a^
mLDFA	89.9 ± 1.2		89.2 ± 1.4	89.6 ± 1.7		88.5 ± 2.7
MPTA	84.0 ± 2.1		91.7 ± 3.4 ^a^	83.7 ± 2.3		92.5 ± 2.4 ^a^
MTPD	−1.1 ± 2.2	−0.9 ± 2.0	−0.8 ± 2.5	−7.4 ± 4.9 ^c^	6.0 ± 2.8 ^a,d^	5.5 ± 2.7 ^a,e^
PPTA	84.2 ± 2.5	82.7 ± 3.6	80.6 ± 4.1 ^a^	82.7 ± 3.2 ^c^	82.1 ± 4.1	81.2 ± 4.3
JLCA	1.4 ± 1.5		1.1 ± 1.0	5.1 ± 1.5 ^c^		0.7 ± 0.9 ^a^
Varus stress angle	5.1 ± 1.1		6.4 ± 2.1 ^a^	7.2 ± 1.7 ^b^		4.0 ± 2.1 ^a,e^
Valgus stress angle	1.9 ± 2.1		0.8 ± 1.8 ^a^	2.3 ± 2.8		0.5 ± 1.3 ^a^
Laxity angle	7.1 ± 2.3		7.1 ± 3.1	9.5 ± 3.2 ^c^		4.5 ± 2.4 ^a,e^

^a^*P* < 0.01 compared to pre-operatively

^b^*P* < 0.01 compared to pre-HTO

^c^*P* < 0.05 compared to pre-HTO

^d^*P* < 0.01 compared to immediately after HTO

^e^*P* < 0.05 compared to post-HTO

In terms of pre- and postoperative comparisons in the HTO group, %MA, HKA, MPTA, and varus-stress angle were increased, and FTA, PPTA, and valgus-stress angle were decreased after surgery (*P* < 0.01). No significant differences in mLDFA, MTPD, JLCA, or laxity angle were seen between before and after surgery. In the TCVO group, lower limb alignment and MPTA were improved and instability was significantly decreased after surgery (*P* < 0.01). While pre- and postoperative mLDFA and PPTA did not differ, the value of MTPD after TCVO was increased and laxity angle and JLCA were markedly decreased relative to the HTO group. Moreover, MTPD and PPTA in both groups did not change from immediately after to 1 year after surgery.

## Discussion

The present results demonstrated that pre-operative KL grade and FTA were higher, and %MA and HKA angle were lower in the TCVO group than that in the HTO group. These findings mean that TCVO can be applied to cases of more advanced knee OA with severe varus deformity, in which the mechanical axis passes relatively medial compared to HTO. Efe et al. [26] reported that a KL grade ≥ 3 is one factor associated with poorer clinical outcomes at an average of 9.6 years after HTO. Some studies have also reported that advanced knee OA and severe malalignment tend to lead to HTO failure [5, 34, 35]. Only one previous study has reported the KL grade of TCVO patients as grade 3 or 4, but the details were not described [27]. Mean %MA and FTA of the TCVO group in the present study were 8.7 ± 13.3% and 183.4 ± 3.9°, respectively. These results suggest that the alignment criteria of TCVO include %MA 5–15%, and FTA 183–186°, as values at which clinical outcomes of HTO are thought to be declined.

In our series, mLDFA values were similar and within normal range in both groups. In a recent case with malalignment of the femoral condyle, we added distal femoral osteotomy (double-level osteotomy) [36]. Most patients with medial knee OA show varus deformity at the proximal tibia (decreased MPTA or MTPD) and knee joint (increased JLCA) [37, 38]. Increasing inclination of the medial tibial plateau is the main contributor to worsened varus deformity [38, 39] and could progress to intra-articular incongruency and lateral thrust phenomenon. Because HTO can manipulate the proximal tibia to a valgus position, the MPTA is corrected, but JLCA and MTPD are not always corrected. In TCVO, shape of the tibial plateau is modified, and the destroyed or inclined medial compartment of the tibial plateau can be restored. In fact, our data confirmed that TCVO can alter not only MPTA, but also JLCA and MTPD, to a greater extent than HTO. The normal range and mean values of JLCA are reported as 0–3° and 1.75°, respectively [40]. Pre-operative JLCA was higher in the TCVO group (5.1 ± 1.5°) than in the HTO group (1.4 ± 1.5°), whereas postoperative JLCAs in both groups were at the same level. In addition, postoperative MTPD in the TCVO group was increased compared to the HTO group. The main concept of TCVO is improvement of femorotibial joint congruity by readjusting the widened lateral joint, as well as realignment of the lower extremity to valgus to shift the mechanical axis laterally. In the medial femorotibial joint of advanced medial knee OA, the medial meniscus and articular cartilage, which fill the gap of the joint space, were considered to be almost torn or completely absent. Postoperative MTPD values in the TCVO group were therefore increased out of necessity to obtain medial joint stability and congruency by bony contact of the articular surface. In addition, postoperative FTA and MPTA values were similar and within normal ranges in both procedures, meaning that alignment of the lower extremity and joint line after TCVO did not interfere with knee function. As a result, TCVO appears better suited to knee OA with a widened lateral femorotibial joint caused by depression or inclination of the medial tibial plateau. Based on our results, TCVO is preferable in cases of knee OA with MTPD within the range of − 10° to − 4°, and JLCA at 4° to 6°.

Previous studies have also indicated that the tibial plateau tends to tilt posteriorly in the sagittal plane after HTO [41, 42] because of insufficient soft-tissue release and inappropriate hinge position [43–45]. Steep PPTA might influence knee kinematics or stability in the anteroposterior direction [46]. The PPTA achieved in the present study indicated that augmentation of posterior tilt in the tibial plateau was avoidable in TCVO.

Although HTO can reportedly improve stability of the knee joint [47, 48], chronic joint instability such as lateral thrust phenomenon remains one of the major factors affecting clinical outcome. The removal of any torn medial meniscus may accelerate progression of joint instability and knee OA [49, 50]. HTO with ligament reconstruction is one surgical option for the treatment of joint laxity [51–53], but requires greater surgical invasion and prolonged rehabilitation and hospitalization [53], in addition to high medical costs. TCVO together with remodeling of the shape of the tibial plateau can improve femorotibial joint congruity and stability. Increased tension in the cruciate ligaments due to making the tibial plateau concave using an L-shaped osteotomy also contributed to increased joint stability. Our results revealed that TCVO could reduce the mean varus stress angle from 7.2° to 4.0°, and laxity angle from 9.5° to 4.5°, without any ligament reconstructions. TCVO is thus desirable for knee OA involving severe joint laxity in the coronal plane. Varus stress angle from 6° to 8°, and laxity angle from 7° to 11° represent potent indicators of TCVO.

Based on the present results, the advantages of TCVO are: 1) correction of varus malalignment of the lower extremity; 2) reconstruction of medial articular deformation of the tibial plateau; and 3) the reduction in joint laxity. Furthermore, 4) early weight-bearing can be started because the osteotomy line does not reach the lateral tibial condyle; 5) risk of hinge fracture is reduced; and 6) reduction of a subluxated lateral joint during the operation is superior to HTO. TCVO is thought to be an effective surgical procedure for patients with advanced varus knee OA, inclined medial tibial plateau, widened lateral femorotibial joint, and high joint instability. However, we need to pay attention to the disadvantages of TCVO. First, correction of the tibia to a valgus position is limited only to the angle at which the lateral joint is reduced. Prudent preoperative planning is required to compare correctable and estimated postoperative %MA. Second, soft-tissue balance cannot be modified directly by this procedure, and therefore medial tightness and lateral laxity may remain after the surgery.

Limitations in this study included the small number of cases and the short duration of follow-up (1 year after surgery). Lee et al. [54] reported that barely any correction loss had occurred from 1 year after HTO, but we must pursue radiographic changes, such as progression of knee OA and correction loss, over the long term after surgery. In addition, detailed clinical outcomes should also be assessed. TCVO with a locking plate and minimally invasive surgical techniques have been introduced since 2008. Further study is therefore warranted to include a large sample size, and a prospective design is needed to better clarify the exact radiological indications, and to determine the clinical availability of TCVO. In the future, demands for osteotomy will increase when regenerative medicine for articular cartilage or meniscus becomes widespread. As the osteotomy is much more cost-effective than TKA [55], the present value of TCVO will be increased as one of the surgical options other than TKAs in the treatment of advanced knee OA.

## Conclusion

We compared the radiological features of HTO and TCVO. TCVO improved %MA, lower limb alignment, tibial morphology to the same extent as HTO. Furthermore, TCVO improved joint laxity and congruity, whereas HTO did not. TCVO appears preferable in cases with advanced knee OA, destroyed or inclined medial tibial plateau, widened and subluxated lateral joint, and high varus-valgus joint instability.

## Data Availability

The datasets used and analyzed during the current study are available from the corresponding author on reasonable request.

## Abbreviations

%MA: Percentage of mechanical axis
ANOVA: Analysis of variance
FTA: Femorotibial angle
HKA angle: Hip-knee-ankle angle
HTO: High tibial osteotomy
JLCA: Joint line convergence angle
KL: Kellgren-Lawrence
MCL: Medial collateral ligament
mLDFA: Mechanical lateral distal femoral angle
MPTA: Medial proximal tibial angle
MTPD: Medial tibial plateau depression
OA: Osteoarthritis
PF: Patellofemoral
PPTA: Posterior proximal tibial angle
ROM: Range of motion
TCVO: Tibial condylar valgus osteotomy
TKA: Total knee arthroplasty
